# Long-term survival outcomes of esophageal squamous cell carcinoma with intraoperative thoracic duct ligation: a large-scale propensity score matching analysis

**DOI:** 10.3389/fonc.2025.1533378

**Published:** 2025-03-05

**Authors:** Ke-xun Li, Si-miao Lu, Chang-ding Li, Cheng-hao Wang, Jia-hua Lv, Qi-feng Wang, Yun-chao Huang, Yong-tao Han, Xue-feng Leng, Lin Peng

**Affiliations:** ^1^ Department of Thoracic Surgery, Sichuan Clinical Research Centre for Cancer, Sichuan Cancer Hospital & Institute, Sichuan Cancer Centre, Affiliated Cancer Hospital of University of Electronic Science and Technology of China (Sichuan Cancer Hospital), Chengdu, Sichuan, China; ^2^ Department of Thoracic and Cardiovascular Surgery, The Third Affiliated Hospital of Kunming Medical University (Yunnan Tumor Hospital), Kunming, China; ^3^ School of Public Health, Chongqing Medical University, Chongqing, China; ^4^ Department of Thoracic Surgery, Zigong First People's Hospital, Zigong, Sichuan, China; ^5^ Department of Radiation Oncology, Sichuan Cancer Hospital & Institute, University of Electronic Science and Technology of China (UESTC), Chengdu, China; ^6^ Radiation Oncology Key Laboratory of Sichuan Province, Chengdu, China

**Keywords:** esophageal squamous cell carcinoma, intraoperative thoracic duct ligation, esophagectomy, overall survival, propensity score matching

## Abstract

**Background:**

Esophagectomy is the primary treatment for localized esophageal squamous cell carcinoma (ESCC). Intraoperative thoracic duct ligation (TDL) has been suggested as an adjunct to reduce the risk of postoperative chylothorax in patients with ESCC, but its effect on long-term oncologic outcomes remains uncertain.

**Methods:**

Data from the Sichuan Cancer Hospital and Institute Esophageal Cancer Case Management Database were analyzed for patients treated between 2010 and 2017. Participants were classified into TDL and non-TDL groups. Univariate Cox regression analyses and propensity score matching (PSM) were used to identify independent risk factors for overall survival (OS).

**Results:**

A total of 2,510 patients were included, with 2,095 in the TDL group and 415 in the non-TDL group. The median follow-up was 63.97 months. No significant differences in OS were observed between the TDL and non-TDL groups (HR: 1.13; 95% CI: 0.96–1.31; P = 0.13). After PSM, the analysis continued to show no significant differences between the groups (P = 0.72).

**Conclusion:**

Intraoperative TDL during esophagectomy did not significantly impact long-term OS in patients with ESCC.

## 1 Introduction

Esophageal cancer is a highly aggressive malignancy with poor overall survival (OS), posing a significant global health challenge. Esophageal squamous cell carcinoma (ESCC) is the predominant histological subtype, particularly in East Asian countries (1, 2). Despite advancements in multimodal treatment strategies centered around esophagectomy (3–6), ESCC remains associated with high rates of lymph node metastasis (LNM) and poor long-term OS (7, 8).

Esophagectomy is the primary curative approach for localized ESCC (9, 10), but long-term outcomes vary based on several factors (11–13), necessitating individualized treatment plans (14–16). However, this procedure carries a notable risk of postoperative complications, such as chylothorax - the leakage of lymphatic fluid into the pleural space- which can lead to severe metabolic and nutritional imbalances, prolonged hospitalization and increased mortality (17, 18).

To mitigate the risk of chylothorax, intraoperative thoracic duct ligation (TDL) has been proposed as an adjunct to esophagectomy. TDL may lower the incidence of chylothorax-related complications (19–21), however, its effect on long-term survival in patients with ESCC remains uncertain, and high-quality studies on this topic are limited in China.

This retrospective cohort study aimed to assess the long-term OS of ESCC patients undergoing esophagectomy with or without intraoperative TDL.

## 2 Materials and methods

### 2.1 Data source

**Figure 1:**
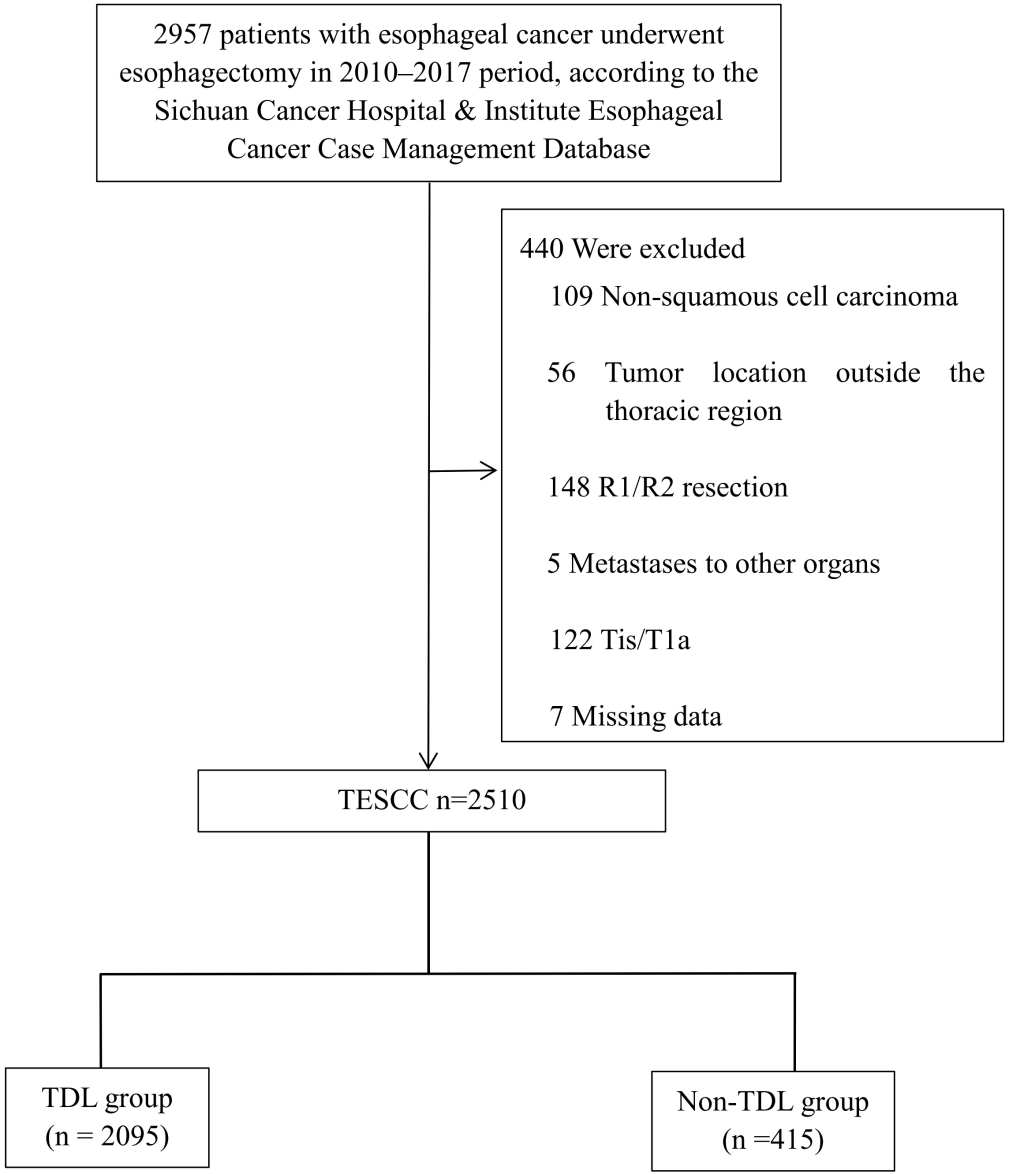
CONSORT diagram of patient selection. TESCC, thoracic esophageal squamous cell carcinoma; TDL, thoracic duct ligation.

Data for this retrospective analysis were collected from the Sichuan Cancer Hospital and Institute Esophageal Cancer Case Management Database (SCCH-ECCM) covering cases from January 2010 to December 2017. The study included patients diagnosed with ESCC who underwent esophagectomy at the institution. Demographic and pathological data were reviewed, and disease staging was conducted according to the AJCC 8th edition TNM system. Patients were followed post-treatment, with assessments every 3 months for the first 2 years and every 6 months for years 3 to 5. OS was calculated from the date of diagnosis to either the last follow-up or death. A total of 2,510 patients with ESCC who underwent esophagectomy between 2010 and 2017 met the inclusion criteria. Criteria excluded patients with non-squamous cell carcinoma, tumors outside the thoracic region, incomplete resection, distant metastasis, early-stage disease, or missing data (**Figure 1**). OS was assessed from diagnosis to the most recent follow-up in March 2021. Smoking history: Patients who reported smoking at least once per day for a duration exceeding one year. Drinking history: Patients who reported consuming alcohol at least once per day for a duration exceeding one year. We hope this clarification addresses your concerns.

Patients were divided into two groups based on intraoperative TDL status. The TDL group included patients who had esophagectomy with concurrent intraoperative TDL, including cases where incidental thoracic duct (TD) injury during surgery required ligation. The Non-TDL group comprised patients who underwent esophagectomy without TDL, where no incidental TD injury occurred, and the duct was preserved intentionally. Clinical outcomes and survival data were compared between groups according to TNM stages as per the AJCC 8th edition.

### 2.2 Ethical statement

This study was conducted in accordance with the Declaration of Helsinki (2013 revision) and approved by the SCCHEC-02-2024-191 Ethics Committee at Sichuan Cancer Hospital.

### 2.3 Statistical analysis

Categorical variables were presented as percentages and compared between the TDL and Non-TDL groups using chi-square or Fisher’s exact tests, as appropriate. The primary outcome of interest was OS), estimated using Kaplan-Meier survival curves and assessed with the log-rank test. To minimize confounding and selection bias, propensity score matching (PSM) was applied. Propensity scores were calculated with a logistic regression model using treatment (TDL vs. Non-TDL) as the dependent variable and relevant covariates, including age, sex and other significant baseline characteristics, as independent variables. A 1:1 nearest-neighbor matching algorithm without replacement was employed, with a calliper width of 0.2 times the standard deviation of the logit of the propensity score. Covariate balance between matched groups was evaluated using standardized mean differences, with values below 0.1 indicating satisfactory balance. Univariable and multivariable Cox proportional hazards regression models were used to identify independent risk factors associated with OS, with hazard ratios (HRs) and 95% confidence intervals (CIs) reported. Sensitivity analyses were conducted using stabilized inverse probability of treatment weighting (IPTW), overlap weighting (OW) and standardized mortality ratio weighting (SMRW) methods to confirm robustness. Adjusted HRs and 95% CIs were calculated with these alternative weighting approaches. All statistical analyses were conducted using SPSS version 23.0 (Chicago, IL, USA) and RStudio with R version 4.3.0. A two-sided P value < 0.05 was considered statistically significant.

## 3 Results

### 3.1 Clinical outcomes

Data from 2,510 patients with ESCC were analyzed retrospectively. As shown in **Figure 1**, 82.1% (2,060/2,510) were male and 17.9% (450/2,510) were female, with approximately 57.5% (1,443/2,510) of cases above stage III (**Table 1**). Among these patients, 2,095 were in the TDL group and 415 were in the Non-TDL group.

**Table 1:** Demographic characteristics of the 2 groups.

Characteristic	Before PSM		*P* value	After PSM		*P* value
	TDL (n= 2095)	Non-TDL (n=415)		TDL (n= 414)	Non-TDL (n= 414)	
Age, years	62 (34-90)	63 (37-85)	<0.001	64 (43-83)	63 (37-85)	0.903
KPS			0.559			0.400
≥90	1179 (56.3%)	240 (57.8%)		228 (55.1%)	240 (58.0%)	
≤80	916 (43.7%)	175 (42.2%)		186 (44.9%)	174 (42.0%)	
Sex			0.433			0.727
Male	1725 (82.3.0%)	335 (80.7%)		330 (79.7%)	334 (80.7%)	
Female	370 (17.7%)	80 (19.3%)		84 (20.3%)	80 (19.3%)	
Smoking			0.096		0.624	
No	1103 (52.6%)	237 (57.1%)		229 (55.3%)	236 (57.0%)	
Yes	992 (47.4%)	178 (42.9%)		185 (44.7%)	178 (43.0%)	
Drinking			<0.001			0.348
No	1572 (75.0%)	269 (64.8%)		256 (61.8%)	269 (65.0%)	
Yes	523 (25.0%)	146 (35.2%)		158 (38.2%)	145 (35.0%)	
Tumor location			0.424			0.387
Upper	500 (23.9%)	87 (21.0%)		94 (22.7%)	87 (21.0%)	
Middle	1136 (54.2%)	231 (55.7%)		240 (58.0%)	231 (55.8%)	
Lower	459 (21.9%)	97 (23.4%)		80 (19.3%)	96 (23.2%)	
Thoracic surgery			<0.001			0.590
MIE	857 (40.9%)	342 (82.4%)		335 (80.9%)	341 (82.4%)	
OE	1238 (59.1%)	73 (17.6%)		79 (19.1%)	73 (17.6%)	
Abdominal surgery			<0.001			0.661
MIE	632 (30.2%)	332 (80.0%)		336 (81.2%)	331 (80.0%)	
OE	1459 (69.6%%)	255 (20.0%)		78 (18.8%)	83 (20.0%)	
Non	4 (0.2%)	0 (0.0%)				
Surgical approach			<0.001			0.710
McKeown	1400 (66.8%)	379 (91.3%)		371 (89.6%)	378 (91.3%)	
Iovr-Lewis	660 (31.5%)	31 (7.5%)		37 (8.9%)	31 (7.5%)	
Sweet	4 (0.2%)	0 (0.0%)				
Left thoracotomy and laparotomy	31 (1.5%)	5 (1.2%)		6 (1.4%)	5 (1.2%)	
Clinical treatment modality			<0.001			0.796
Preoperative CT or RT/CRT plus surgery	44 (2.1%)	2 (0.5%)		2 (0.5%)	2 (0.5%)	
Surgery alone	996 (47.5%)	273 (65.8%)		283 (68.4%)	272 (65.7%)	
Surgery plus postoperative CT or RT/CRT	1055 (50.4%)	140 (33.7%)		129 (31.2%)	140 (33.8%)	
Number of RLNs	20 (0-97)	20 (1-65)	0.728	20 (0-77)	20 (1-65)	0.723

TDL, thoracic duct ligation; CRT, chemoradiotherapy; CT, chemotherapy; MIE, minimally invasive esophagectomy; OE, open esophagectomy; RT, radiotherapy.

### 3.2 Recurrence and short-term outcomes

Further analyses include recurrence in the upper mediastinum, recurrence in the residual esophagus, anastomotic recurrence, distant organ and bone metastasis, lymph node (LN) recurrence in the mediastinal and supraclavicular zones, as well as recurrence and metastasis in the abdominal organs, peritoneal space, peritoneum, and abdominal LNs. Additionally, mortality rates at 30 days, 90 days, and six months were incorporated into **Table 2**. The outcomes indicated that patients in the TDL group exhibited significantly higher rates of recurrence in the upper mediastinum (P=0.013) and LN recurrence in the mediastinal and supraclavicular zones (P=0.041) compared to those in the Non-TDL group. However, for the remaining outcome measures, no statistically significant differences were observed between the two groups. After PSM to mitigate potential confounding factors, we found that there were no statistically significant differences in any of the recurrence and metastasis indicators between the TDL and Non-TDL groups. This suggests that while the TDL group exhibited higher rates of certain types of recurrence initially, these differences were not sustained when accounting for baseline characteristics through PSM.

**Table 2:** Outcomes post-esophagectomy in 2 groups.

Characteristic	Before PSM		*P* value	After PSM		*P* value
	TDL (n= 2095)	Non-TDL (n=415)		TDL (n= 414)	Non-TDL (n= 414)	
Tumor grade			0.168			0.848
Well G1	392 (18.7%)	63 (15.2%)		60 (14.5%)	63 (15.2%)	
Moderate G2	860 (41.1%)	186 (44.8%)		181 (43.7%)	186 (44.9%)	
Poor or undifferentiated G3	843 (40.2%)	166 (40.0%)		173 (41.8%)	165 (39.9%)	
Lymphovascular invasion			0.118			0.567
No	1715 (81.9%)	353 (85.1%)		346 (83.6%)	352 (85.0%)	
Yes	380 (18.1%)	62 (14.9%)		68 (16.4%)	62 (15.0%)	
Nerve invasion			0.090			0.621
No	1701 (81.2%)	322 (77.6%)		315 (76.1%)	321 (77.5%)	
Yes	394 (18.8%)	93 (22,4%)		99 (23.9%)	93 (22.5%)	
Pathological T stage			<0.001			0.657
T1	158 (7.5%)	44 (10.6%)		39 (9.4%)	44 (10.6%)	
T2	396 (18.9%)	113 (27.2%)		117 (28.3%)	112 (27.1%)	
T3	1345 (64.2%)	239 (57.6%)		245 (59.2%)	239 (57.7%)	
T4	196 (9.4%)	19 (4.6%)		13 (3.1%)	19 (4.6%)	
pN			0.016			0.799
N0	887 (42.3%)	197 (47.5%)		197 (47.6%)	196 (47.3%)	
N1	628 (30.0%)	133 (32.0%)		123 (29.7%)	133 (32.1%)	
N2	388 (18.5%)	62 (14.9%)		66 (15.9%)	62 (15.0%)	
N3	192 (9.2%)	23 (5.5%)		28 (6.8%)	23 (5.6%)	
TNM stage			0.051			0.999
I	158 (7.5%)	44 (10.6%)		44 (10.6%)	44 (10.6%)	
II	711 (33.9%)	154 (37.1%)		151 (36.5%)	153 (37.0%)	
III	969 (46.3%)	183 (44.1%)		185 (44.7%)	183 (44.2%)	
IV	257 (12.3%)	34 (8.2%)		34 (8.2%)	34 (8.2%)	
Recurrence in the upper mediastinum	219 (10.5%)	27 (6.5%)	0.013	27 (6.5%)	27 (6.5%)	1.000
Recurrence in the residual esophagus	226 (10.8%)	35 (8.4%)	0.151	37 (8.9%)	35 (8.5%)	0.805
Anastomotic recurrence	17 (0.8%)	2 (0.5%)	0.691	2 (0.5%)	2 (0.5%)	1.000
Lymph node recurrence in the mediastinal and supraclavicular zones	468 (22.3%)	74 (17.8%)	0.041	74 (17.9%)	74 (17.9%)	1.000
Distant organ, bone and abdominal lymph nodes	366 (17.5%)	63 (15.2%)	0.258	69 (16.7%)	63 (15.2%)	0.569
Died in 30 days	11 (0.5%)	5 (1.2%)	0.211	1 (0.2%)	5 (1.2%)	0.219
Died in 90 days	40 (1.9%)	8 (1.9%)	0.980	7 (1.7%)	8 (1.9%)	0.794
Died in half a year	109 (5.2%)	22 (5.3%)	0.934	21 (5.1%)	22 (5.3%)	0.876

### 3.3 Recurrence and short-term outcomes

**Figure 2:**
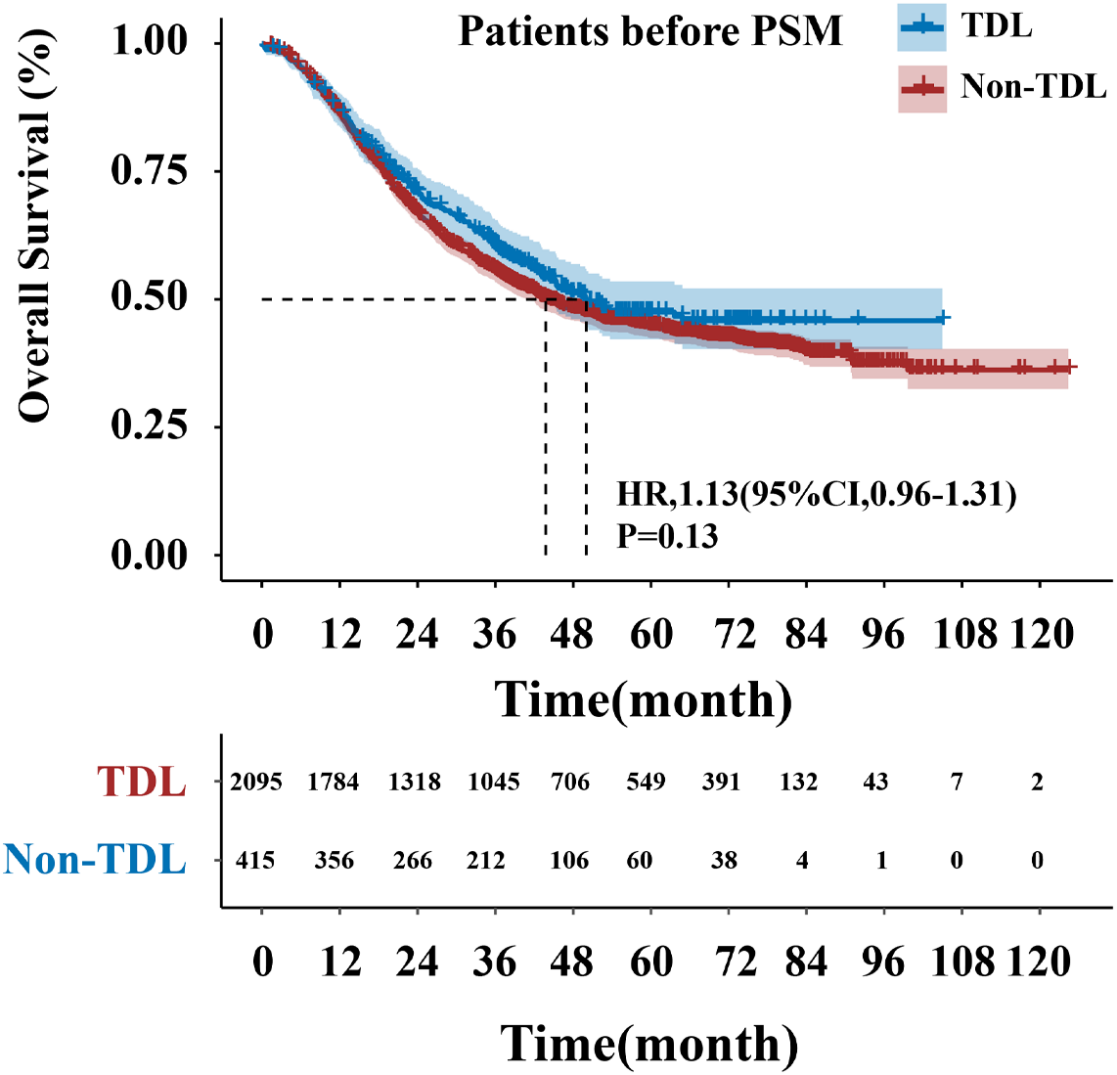
Overall survival curve of TDL and Non-TDL groups.

**Figure 3:**
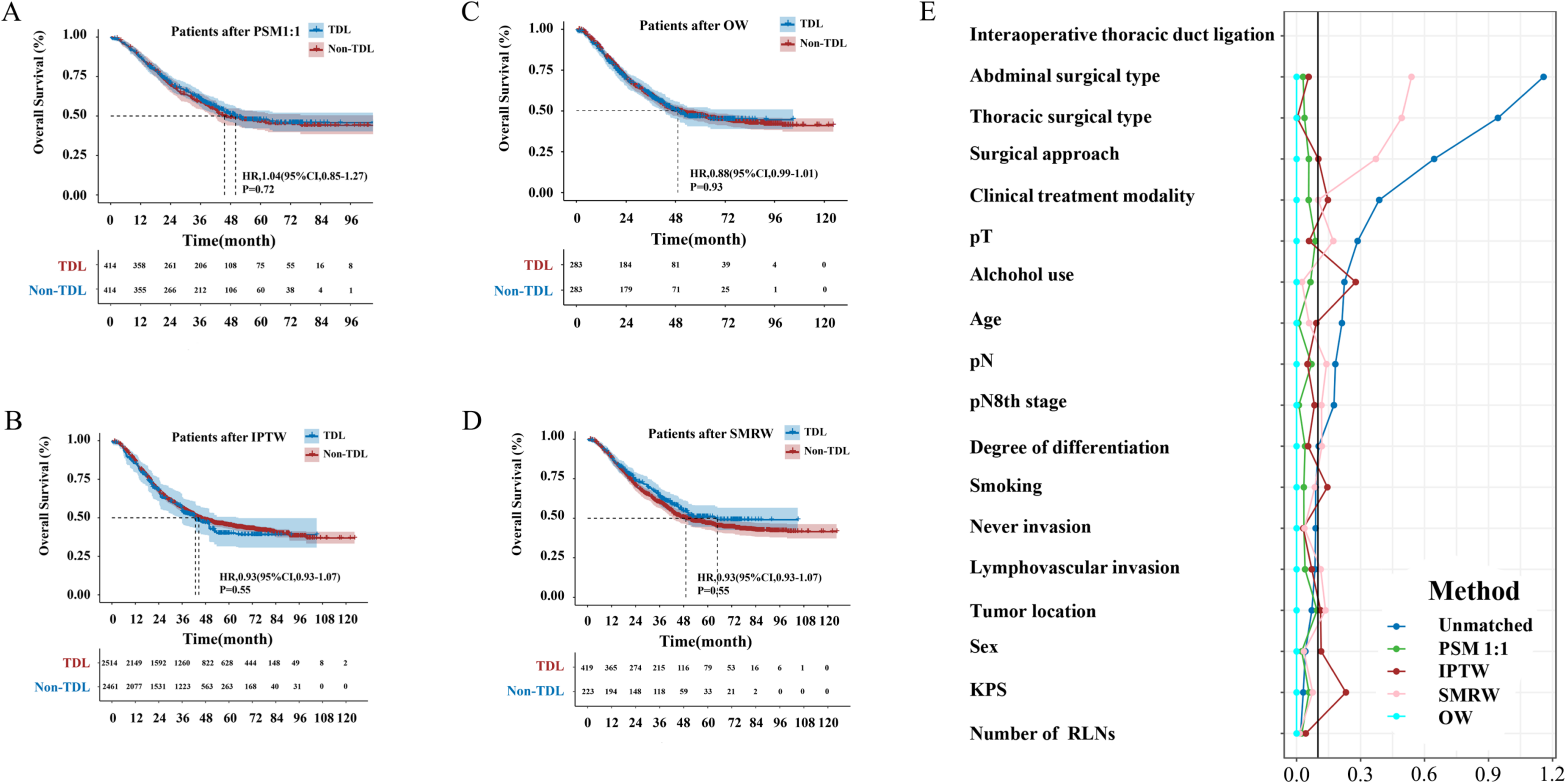
**(A)** Overall survival curves of TDL and Non-TDL groups after PSM; **(B)** Overall survival curves of TDL and Non-TDL groups after IPTW; **(C)** Overall survival curves of TDL and Non-TDL groups after OW; **(D)** Overall survival curves of TDL and Non-TDL groups after SMRW; **(E)** Standardized in the subjects stratified by characteristic.

The median follow-up period was 63.97 months. The TDL group had a median OS of 43.80 months (95% CI: 38.83–48.77), while the Non-TDL group had a median OS of 50.03 months. The 1-, 3-, and 5-year OS rates were 86%, 56% and 45% for the TDL group, and 87%, 60% and 47% for the Non-TDL group (HR: 1.13; 95% CI: 0.96–1.31; P = 0.13; **Figure 2**). Post-PSM, no significant differences were observed between groups (HR: 0.96; 95% CI: 0.96–1.04; P = 0.72; **Figure 3A**). Similar results were obtained with IPTW, OW and SMRW methods (HR: 0.93; 95% CI: 0.93–1.08; P = 0.55; **Figure 3B**; HR: 0.88; 95% CI: 0.99–1.01; P = 0.93; **Figure 3C**; and HR: 1.14; 95% CI: 0.88–1.14; P = 0.19; **Figure 3D**). **Figure 3E** illustrated that for 1:1 PSM, IPTW, and OW methods, the standardized mean difference for all variables is less than 0.02.

### 3.4 Risk factors

**Figure 4:**
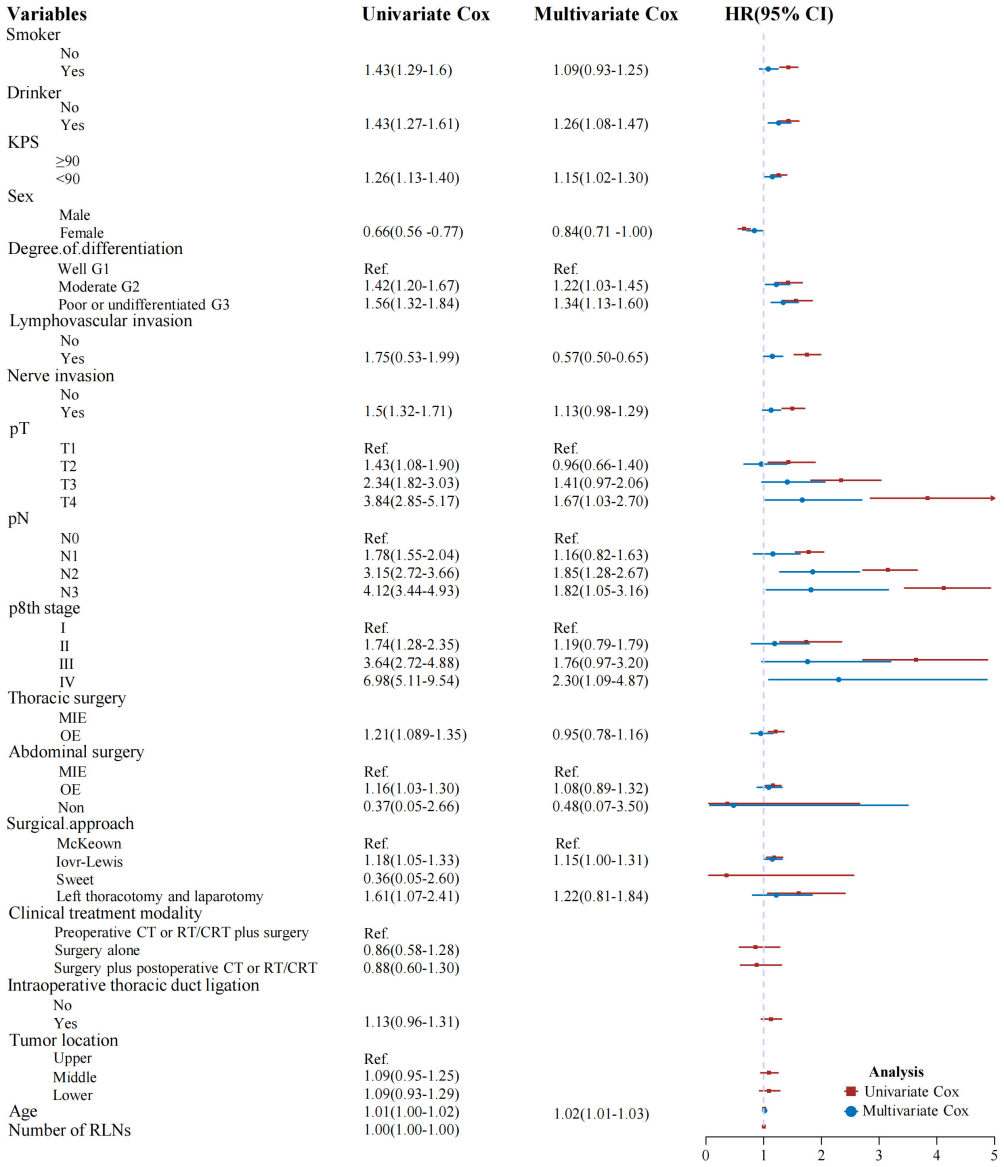
Univariate and multivariate Cox regression analyses of factors affecting patient survival.

Univariate analysis identified several factors significantly impacting OS after esophagectomy: alcohol use (P < 0.001), smoking status (P < 0.001), age (P = 0.004), KPS scores (P < 0.001), sex (P < 0.001), thoracic and abdominal surgical type (P < 0.001 and P = 0.024, respectively), surgical approach (P = 0.004), tumor grade (P < 0.001), lymphovascular and nerve invasion (P < 0.001 each), pathological T and N categories (P < 0.001) and TNM stage (P < 0.001; **Figure 4**). Multivariate analysis indicated that alcohol use (P = 0.003), age (P < 0.001), KPS scores (P = 0.022), tumor grade (P = 0.003), lymphovascular invasion (P = 0.047) and pathological T and N categories (P < 0.001 each) were independently associated with OS post-esophagectomy (**Figure 4**).

## 4 Discussion

This retrospective cohort study investigated the impact of TDL on the long-term OS of patients with ESCC who underwent esophagectomy. While previous research has indicated the potential benefits of TDL in minimizing chylothorax incidence, our findings did not reveal a significant difference in OS between patients who underwent TDL and those who did not. This suggests that the role of TDL in enhancing long-term survival outcomes in ESCC treatment may be limited. Although TDL theoretically reduces chylothorax-related complications, the lack of significant disparity in OS between the TDL and Non-TDL groups indicates that TDL may not substantially affect long-term prognosis (19–21). This conclusion held even after rigorous statistical adjustments using PSM and various weighting methods, indicating that the decision to perform TDL may not influence survival outcomes in patients with ESCC. The study further identified several independent factors affecting OS post-esophagectomy, including alcohol use, smoking, age, KPS scores, sex, surgical approach, tumor grade, lymphovascular invasion, nerve invasion and TNM stage.

The absence of a significant difference in OS between the two groups underscores the complexity of factors impacting survival outcomes in ESCC. While surgical innovations aim to reduce perioperative risks and enhance short-term outcomes, long-term survival is more intricately linked to factors such as tumor stage, patient health, genetic predispositions and the effectiveness of adjunctive treatments like chemotherapy, radiotherapy and immunotherapy (22–25).

Before PSM, patients in the TDL group indeed exhibited higher rates of upper mediastinal recurrence (P=0.013) and mediastinal/supraclavicular LN recurrence (P=0.041). We hypothesize that this discrepancy may be attributed to baseline imbalances in TNM staging between the two cohorts before matching. Specifically, the TDL group showed a tendency toward more advanced disease stages at initial diagnosis, which could predispose this population to increased locoregional recurrence risk. Importantly, these differences were mitigated after PSM adjustment, suggesting that tumor stage rather than TDL status itself may be the primary driver of recurrence patterns.

The current treatment paradigm for ESCC primarily focuses on a multimodal approach centered around surgery, with additional neoadjuvant therapies like radiotherapy, chemotherapy and immunotherapy. This is followed by adjuvant chemotherapy or immunotherapy for maintenance (26–29). Lymphadenectomy and TDL are crucial components of the surgical procedure. Lymphadenectomy is essential for accurate staging and potentially curative treatment, as LN involvement is a key prognostic factor in ESCC. Comprehensive LN removal allows for precise staging and may eradicate micrometastatic disease, thereby enhancing survival (30–32). While TDL is effective in reducing chylothorax incidence, its impact on long-term survival remains inconclusive (19–21). Additional large-scale, multicenter studies are warranted to establish the role of TDL in ESCC treatment, especially concerning long-term survival, to better inform surgical decisions and improve patient outcomes.

A study in Japan investigated the impact of the TD and surrounding LN resection on short-term and long-term outcomes, as well as postoperative nutritional status, in patients with esophageal cancer undergoing esophagectomy. Among the 145 patients (27.0%) who underwent TD and surrounding LN resection (33), those in the resection group had more advanced clinical stages and more frequently received preoperative treatment. Additionally, these patients experienced longer surgical time and greater intraoperative blood loss. Complications classified as Clavien–Dindo Grade II, along with pulmonary complications, were more prevalent in the resection group. Multivariate analysis identified TD resection as an independent risk factor for pulmonary complications and indicated a possible association with Clavien–Dindo Grade II complications. Despite a higher count of thoracic anatomical LNs resected in the resection group, OS rates remained similar across all stages regardless of TD resection. Furthermore, one year postoperatively, the nutritional status between the groups was comparable. The findings suggest that extensive LN resection combined with TD resection may not improve prognostic outcomes and could increase postoperative complications, potentially affecting survival negatively (33). Extended LN dissection alongside TD resection may correlate with higher rates of chylothorax and other complications (33–35). It was noted that chylothorax, in particular, can delay chemotherapy initiation, potentially diminishing the survival benefits of aggressive resection.

In 2023, a national study in Japan further analyzed TD resection outcomes using data from the Comprehensive Registry of Esophageal Cancer in Japan, including 12,237 patients who underwent esophagectomy between 2007 and 2012. Results showed no significant OS improvement in patients who had TD resection compared to those who did not. Interestingly, while the TD resection group had a higher retrieval of mediastinal nodes and lower rates of LN recurrence, this did not translate to better survival outcomes. On the contrary, these patients had an increased incidence of distant metastasis. This finding challenges the assumed preventive benefit of TD resection in esophageal cancer, suggesting that this approach may elevate the risk of distant metastasis despite its theoretical advantages in LN management. The increased retrieval of mediastinal nodes in the TD resection group, although indicating a more thorough surgical clearance, did not correlate with enhanced survival. This observation underscores the complex relationship between the extent of surgical intervention and prognosis in esophageal cancer. Hou and colleagues’ study provided valuable insights by categorizing patients into those who underwent prophylactic TDL and those who did not, allowing for a clear comparison of outcomes. Contrary to the expected benefits, there was no significant difference in the incidence of postoperative chylothorax between the two groups. This challenges the commonly held rationale for TDL, which is often performed to prevent this specific complication. Similarly, Aiolfi et al.’s meta-analysis reinforces these findings, highlighting the ongoing debate surrounding the efficacy of TDL in esophageal cancer surgeries. By analyzing data from a substantial patient cohort, the meta-analysis showed that TDL does not significantly reduce the risk of postoperative chylothorax, thus questioning the procedure’s protective role. Moreover, both studies consistently indicate a concerning impact of TDL on long-term survival, with patients undergoing the procedure experiencing reduced overall survival compared to those who did not. These findings collectively call into question the routine implementation of prophylactic TDL in esophagectomy. Rather than uniformly benefiting patients, TDL may inadvertently compromise long-term survival outcomes (34–38).

This study has several limitations. As a retrospective analysis, it is subject to inherent biases, including selection bias and uncontrolled confounding factors. Although PSM was employed to reduce these biases, residual confounders may still be present. Furthermore, the single-institution setting limits the generalizability of the findings to other populations or healthcare systems. Multicenter studies with larger sample sizes are essential to confirm these results. Additionally, this study focused solely on the impact of TDL on long-term OS, without assessing other critical outcomes such as disease-free survival or quality of life. These outcomes are important for a holistic evaluation of TDL’s effectiveness. The significance of preoperative therapy, especially in patients with T2/T3N0-1 esophageal squamous cell carcinoma, has been acknowledged in the CROSS study. Even the results of CROSS and NEOCRTEC5010 studies were used as guidelines to recommend preoperative neoadjuvant therapy for patients with locally advanced esophageal cancer (4, 35), China’s own data of esophageal squamous cell carcinoma (NEOCRTEC5010 study) was published in 2018 and the first guideline of Chinese Society Of Clinical Oncology (CSCO) on diagnosis and treatment of esophageal cancer published in 2019. In the data we included from 2010 to 2017, real-world clinical practice primarily involved surgery alone for resectable tumors. However, over the past 5 years, the proportion of neoadjuvant therapy has gradually increased in our center and China. In future studies, we will consider including more patients who have undergone neoadjuvant therapy and investigate the potential impact of such treatments on patient OS outcomes. Future research should include a broader range of outcomes to provide a more comprehensive assessment of TDL’s impact.

## 5 Conclusions

This retrospective cohort study found no statistically significant difference in long-term OS between patients with ESCC who underwent esophagectomy with intraoperative TDL and those who did not. While locoregional control remains important, the increased risk of complications—especially those affecting timely systemic therapy—may offset theoretical oncologic advantages. This supports the recommendation for individualized surgical strategies prioritizing both short-term recovery and long-term survival.

## Data Availability

The raw data supporting the conclusions of this article will be made available by the authors, without undue reservation.
